# Boost Protein Expression through Co-Expression of LEA-Like Peptide in *Escherichia coli*

**DOI:** 10.1371/journal.pone.0082824

**Published:** 2013-12-12

**Authors:** Shinya Ikeno, Tetsuya Haruyama

**Affiliations:** Department of Biological Functions and Engineering, Kyushu Institute of Technology, Kitakyushu Science and Research Park, Kitakyushu, Fukuoka, Japan; University of Crete, Greece

## Abstract

The boost protein expression has been done successfully by simple co-expression with a late embryogenesis abundant (LEA)-like peptide in *Escherichia coli*. Frequently, overexpression of a recombinant protein fails to provide an adequate yield. In the study, we developed a simple and efficient system for overexpressing transgenic proteins in bacteria by co-expression with an LEA-like peptide. The design of this peptide was based on part of the primary structure of an LEA protein that is known hydrophilic protein to suppress aggregation of other protein molecules. In our system, the expression of the target protein was increased remarkably by co-expression with an LEA-like peptide consisting of only 11 amino acid residues. This could provide a practical method for producing recombinant proteins efficiently.

## Introduction

The expression and production of recombinant proteins is a key technology in various fields of research and development. For practical reasons, and in the light of recent development of peptide/protein drugs (biologics), attention has focused particularly on the efficiency of production of recombinant protein, and various host cells have been examined with the aim of achieving efficient production of such protein [1],[2],[3],[4],[5]. *Escherichia coli* is widely used in producing a variety of recombinant proteins, because the genetic engineering of this bacterium is among the best understood and developed of all organisms [3],[4]. For recombinant protein, high levels of production are required. If a technique could be developed to eliminate disturbances in protein expression, this might provide a solution that would permit the large-scale production of recombinant proteins. Many previous studies have been made with the aim of developing efficient techniques for the production of biologically active protein. The approaches including optimization of culture condition [4],[5], co-expression of molecular chaperones [3], and the used of solubility tags such as maltose binding protein (MBP) or glutathione S-transferase (GST) [4] are often used for achieving efficient protein production. These can be effective approaches in conventional protein production. But the genetically method requires the removal of the large tag from either the expressed target proteins or the introduction of a number of the chaperone gene into the host cell. Therefore, the simple and efficient system for enhanced protein expression are required in various fields of research and development.

Late embryogenesis abundant (LEA) proteins are a group of the hydrophilic proteins that are found among the families of proteins that are expressed for anhydrobiosis both in plants [6],[7],[8], and in animals [8],[9],[10],[11]. Although a variety of LEA proteins exist, all show has hydrophilic character [12]. Several studies have reported on improvements in the solubility of protein expressed in cell body achieved by utilizing the hydrophilic characteristic of LEA proteins [13],[14],[15]. Singh *et al.* developed a technique for facilitating recombinant expression of recalcitrant proteins by using LEA protein as fusion partners [13]. They found that fusion of the LEA protein provides sufficient solubility to permit overexpression of hydrophobic protein in *E.coli*. In this system, however, the large protein tag has to be removed from the expressed target protein by digestion with a protease as in the case of MBP and GST fusion protein. Chakrabortee *et al.* reported that proteins can be stabilized both *in vitro* and *in vivo* by co-expression of AavLEA1 from the nematode *Aphelenchus avenae* [14]. The co-expressed the LEA protein showed anti-aggregation activity, thereby demonstrating the presence of co-expression with polyglutamine or polyalanine-expansion proteins *in vivo*. However, the expressed LEA proteins cause difficulties in purification when used in practical production of proteins.

LEA proteins are classified into six groups on the basis of expression pattern and sequence similarities [16]. Group 3 LEA proteins have been identified in plant and animals [11],[17],[18],[19]. These proteins are characterized by the structural feature of a repeating sequence of 11 amino acids [20]. The LEA forms a random coil in solution at normal temperature, but exhibits an α-helical component and forms a superhelical structure under dry condition [21],[22],[23]. Furthermore, an artificial peptide with a design based on the LEA protein motif also shows the same conformational change under dry conditions *in vitro* [24].

In order to solve the problem of the efficient expression of protein, we pay attention into the hydrophilic and anti-aggregation property of LEA proteins. We hypothesized that, by taking advantage of these properties of the LEA protein, LEA protein-motif peptide (LEA-like peptide) might play a similar role in anti-aggregation of expressed protein within the cell and enhance the protein expression.

In this study, we attempted to express the target protein together with LEA-like peptide in *E.coli*, with the aim of developing a co-expression system that would permit efficient production of target protein. We also discussed about whether LEA-like peptide improved the transcription activity or the folding activity of the expressed protein within the cell.

## Materials and Methods

### Design of LEA-like peptide

The design of the LEA-like peptide used in this study was based on that of three LEA proteins (PvLEA1, PVLEA2 and PvLEA3) derived from *Polypedilum vanderplanki*. A sequence frequently found in these LEA proteins was identified, and the peptide Ala-Lys-Asp-Gly-Thr-Lys-Glu-Lys-Ala-Gly-Glu was selected as the repeating unit of the LEA-like peptide.

### Plasmid construction

pRSFDuet-1 (Novagen) was used for the co-expression target proteins (GFP, CAT, GUS, and HFBII) and the LEA-like peptide. HFBII is expressed as a fusion protein with GFP for readily evaluation of the protein expression. The vector contains two multiple cloning sites (*MCS1*, *MCS2*), each of which is preceded by a *T7 lac* promoter and a ribosome binding site. The gene for GFP and LEA-like peptide were subcloned to *MCS1* and *MSC2*, respectively. The GFP gene was subcloned into the BamHI and HindiIII site in MCS1 of pRSFDuet-1. The restriction sites BamHI and HindiIII were introduced on either side of the gene encoding the GFP by PCR using the following pair of primers: Fw-BamHI-GFP (CGGGGATCCATGGCTAGCAAAGGAGAAGAA) and Rev-HindIII-GFP (GCCAAGCTTTCAGTTGTACAGTTCATCCAT). The PCR reaction mixture (50 µl) contained 100 ng of template plasmid pQBI63 (Takara), 100 pmol each primer, 2.5 U Primestar Polymerase (Takara), 10 nmol a dNTP mixture, and 10 µl of the appended 5× reaction buffer. PCR was performed as follows; 1 cycle of 94°C for 2 min; 30 cycles of 94°C for 15 s, 55°C for 30 s, and 68°C for 1 min; and then 1 cycle of 68°C for 10 min. The amplified DNA fragment was digested by BamHI and HindIII, purified by agarose gel electrophoresis, and ligated into the digested pRSFDuet-1 vector. CAT, GUS and GFP-HFBII gene were subcloned into the digested pRSFDuet-1 vector in the same way.

The subcloned vectors were treated with NdeI and XhoI, and ligated small DNA fragment that was hybridized following two oligo-DNA units: 5'-TATGGATATCTAAC-3' and 5'-TCGAGTTAGATATCCA- 3'.

The In-Fusion Cloning Kit (Clontech) was used to subclone the repeated LEA-like peptide gene into the expression vector. Following each oligo-DNA pairs, In-F LEA-A (5'-GATATACATATGGATGCGAAAGACGGGACG-3' and 5'-CTTTCGTCCCGTCTTTCGCATCCATATGTATATC-3') In-F LEA-B (5'-AAAGAAAAAGCAGGAGAAGCGAAAGACGGGACG-3' and 5'-CTTTCGTCCCGTCTTTCGCTTCTCCTGCTTTTT-3'), and In-F LEA-C (5'-AAAGAAAAAGCAGGAGAAATCTAACTCGAGTCT-3' and 5'-AGACTCGAGTTAGATTTCTCCTGCTTTTT-3') were hybridized in Tris-HCl buffer (pH 8.0) containing 50 mM KCl. The hybridized DNA LEA-B was phosphorylated at 37°C for 1 h by using T4 Polynucleotide Kinase (Takara), followed by treatment at 65°C for 20 min to deactivate the enzyme. LEA-A, LEA-C and phosphorylated LEA-B were ligated by using a DNA Ligation Kit (Takara) at 17°C for 2 h. The ligated DNA fragments were purified by agarose gel electrophoresis, and subcloned into the EcoRV-digested expression vector at 37°C for 15min by using In-Fusion Cloning Kit, followed by treatment at 50°C for 15 min. The construction of all plasmid vectors were verified by DNA sequencing.

Expression vectors for LEA- II through LEA-V were constructed to subclone each of the corresponding genes into the EcoRV site of expression vector. The following oligo-DNA pairs encoded the LEA-like peptides; LEA-II (5'-GCGAAAGACGGGCTGAAAGAAAAAGCAGGAGAA-3' and 5'-TTCTCCTGCTTTTTCTTTCAGCCCGTCTTTCGC-3'), LEA-III (5'-GCGGGAGACGGGCTGGGAGAAGGAGCAGGAGAA-3' and 5'-TTCTCCTGCTCCTTCTCCCAGCCCGTCTCCCGC-3'), LEA-IV (5'-GCGAAAGGAGGGCTGAAAGGAAAAGCAGGAGGA-3' and 5'-TCCTCCTGCTTTTCCTTTCAGCCCTCCTTTCGC-3') and LEA-V (5'-TCCAAAGACGGGACGAAAGAAAAAAGCGGAGAA-3' and 5'-TTCTCCGCTTTTTTCTTTCGTCCCGTCTTTGGA-3'). The oligo-DNAs were phosphorylated at 37°C for 1 h by using T4 Polynucleotide Kinase, followed by treatment at 65°C for 20 min. Each pair was then hybridized in Tris-HCl buffer (pH 8.0) containing 50 mM KCl. The hybridized DNA fragments were subcloned into EcoRV-digested the expression vector at 37°C for 1 h by using DNA ligase (Takara). The constructed of the expression vector was verified by DNA sequencing. All the origo-DNAs were obtained from Genenet (Fukuoka).

### Co-expression of recombinant protein and LEA-like peptide

The transformed *E. coli* BL21 (DE3) containing the plasmid for co-expression of LEA-like peptide and target protein were cultured in Luria Bertani (LB) medium containing kanamycin (50 µg/mL) at 37°C for 12 h. The preculture medium was then seeded into the incubated LB medium, and the culture was continued at 37°C. When the absorbance at 600 nm reached 0.5, IPTG was added as an inducer to the culture medium at a final concentration of 0.1 mM. After induction, cultivation was continued at 37°C. The cultivated microorganism was harvested by centrifugation at 3000 rpm for 10 min, and suspended in 0.1 M phosphate buffer (pH 7.4). GFP fluorescence of *E.coli* was detection by fluorescence spectrometry on an FP-6600 instrument (JASCO) with excitation at 488 nm and measurement at an emission wave length of 508 nm. The quantitative determination of CAT was performed with colorimetric enzyme immunoassay (Rhoche). The expression of GUS was evaluated qualitatively by using 5-bromo-4-chloro-3-indolyl glucuronide (X-Gluc) as the indigogenic substrate.

### Tricine-SDS PAGE analysis

In order to extract the soluble protein, SoluLyse (Genlantis) was added into the bacterial pellet and gently mix for 10 minutes. After the reaction, the insoluble fraction was removed by centrifugation. The soluble sample solution was mixed with an equal volume of the sample buffer (2x containing 125 mM Tris-HCl (pH 6.8), 4% SDS, 20% glycerol, 0.1 mg/ml of bromophenol blue, and 10% 2-sulfanylethanol). The mixture was heated at 95°C for 5 min then cooled on ice for 5 min. The prepared samples were loaded into the well of a 15% polyacrylamide gel and separated by SDS electrophoretic techniques with tricine-Tris buffer. After electrophoresis, the separated proteins were visualized by staining with Coomassie Brilliant Blue.

### m-RNA analysis (extract of total RNA, reverse transcription and real-time PCR)

Total RNA was isolated from the RNAprotect-stabilized *E. coli* cultures using the RNeasy Protect Bacteria Kit (Qiagen). Cultured E. coli were mixed and incubated with RNAprotect Bacteria Reagent to stabilize of the RNA in bacterial cultures. The bacteria cell wall was digested enzymatically by a lysozyme, and the extracted total RNA was purified on an RNeasy Mini column (Qiagen). The extracted total RNA was reverse-transcribed to cDNA by using the PrimeScript RT enzyme (Takara). cDNA synthesis was performed on a 10-µl scale in mixture of 2 µl 2x RT buffer, 0.5 µl RT enzyme mix, 25 pmol oligo dT primer, 50 pmol random 6mers primer, and 2 µl extracted total RNA. The reaction was performed at 37°C for 15 min. The mRNA was quantified by mean of real-time PCR. Real-time PCR reactions were carried out by using a Step One real-time PCR system (Applied Biosystems) and SYBR Premix Ex Taq II (Takara). The sequences of the primer sets were S-GFP 506 (5'-GCCACAACATTGAAGATGGAAG-3') and AS-GFP 685 (5'-CAGCAGCTGTTACAAACTCAAG-3') for quantification of GFP mRNA, and f1L (5'- GAGTTTGATCCTGGCTCAG-3') and r1L (5'- GTATTACCGCGGCTGCTGG-3') for endogenous control. Each 20 µl of reaction mixture contained 4 pmol of each primer, 10 µl 2× SYBR Premix Ex Taq II, 0.4 µl ROX Reference Dye (Takara), and 2 µl cDNA. Real-time PCR was performed at 95°C for 30 s, followed by 40 cycles of 95°C for 5 s, 55°C for 20 s and 72°C for 30 s. The threshold cycle (Ct) for unknown samples was determined by using the Step One software (Applied Biosystems), and the relative expression levels of GFP mRNA were calculated by means of standard-curve method by using 16S ribosomal RNA as an endogenous control.

## Results and Discussion

### Co-expression of green fluorescent protein with an LEA-like peptide

**Figure 1:**
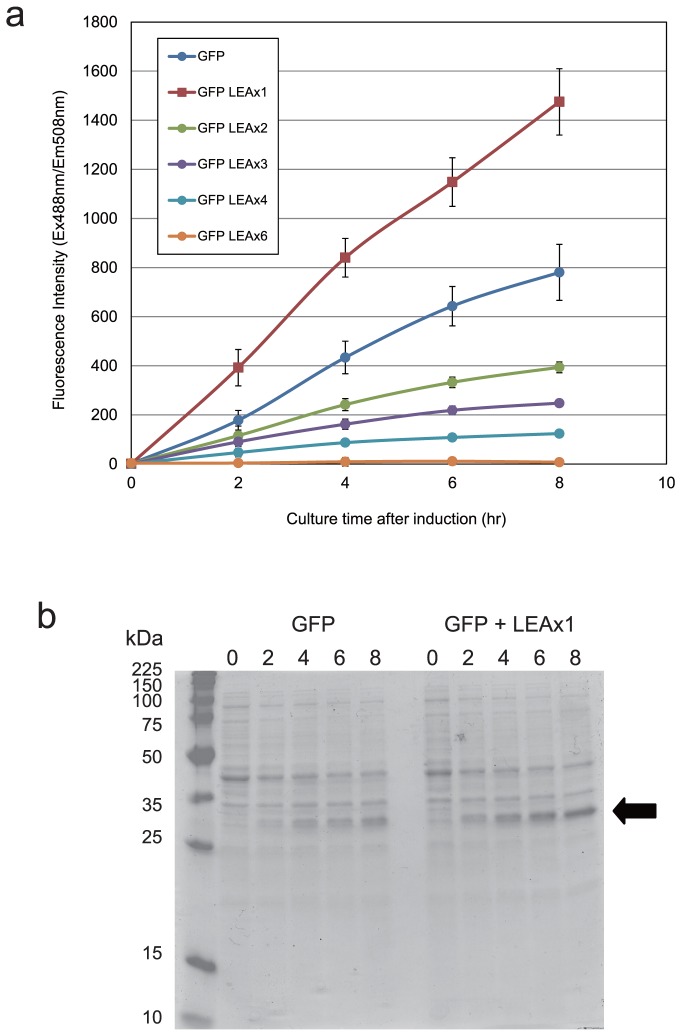
Effect on GFP expression of co-expression with LEA-like peptide in *E.coli.* (a) Time dependence of GFP expression after IPTG induction (b) The GFP expression with or without 11amino acids LEA-like peptide were analyzed by SDS-PAGE analysis.

We selected Green Fluorescent Protein (GFP) as a target protein for cellular expression because the evaluation of the expression is very simple to detect only the fluorescence without extracting it from cell. Figure 1a shows time course of GFP fluorescence with co-expression of each of several LEA-like peptide containing between one and six repeating 11-amino acid units after isopropyl β-D-1-thiogalactopyranoside (IPTG) induction. The GFP fluorescence was effectively increased only when 11amino acids LEA-like peptide itself was co-expressed in the cell. In contrast, fluorescence intensity decreased when peptides of greater lengths were co-expressed. The intensity decreased with increasing peptide length and fell to almost zero when a peptide containing six repeating 11-amino acid units was co-expressed. The protein expression with or without 11amino acids LEA-like peptide was examined by mean of SDS-PAGE (Figure 1b). GFP expression was effectively enhanced by co-expression with the LEA-like peptide. These results support our hypotheses that LEA-like peptide can enhance the transcription activity or folding rates of the protein.

### Analysis of mRNA transcription by means of real-time polymerase chain reaction

**Figure 2:**
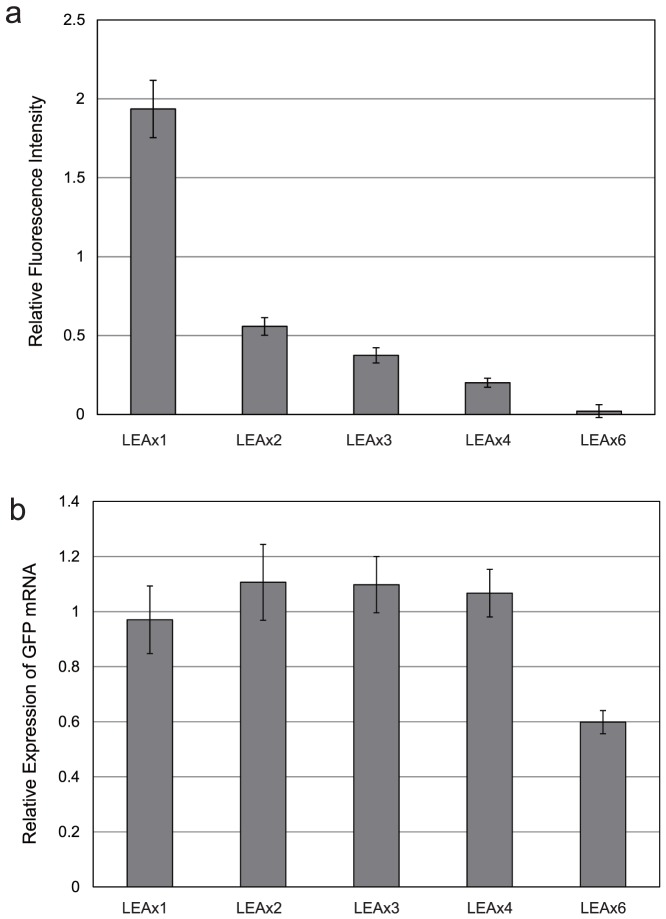
Relative GFP expression and mRNA, with co-expression of each repeated LEA-like peptide 4 h after induction. (a) Relative GFP expression levels and (b) mRNA levels with co-expression of each repeated LEA-like peptide 4 h after induction. The co-expressed LEA-like peptide were as follows: 1 repeat, 2 repeats, 3 repeats, 4 repeats and 6 repeats.

To analyze the transcriptional activity when the LEA-like peptide was co-expressed, the relative abundances of GFP mRNA 4 h after induction were analyzed by means of real-time polymerase chain reaction with SYBR Green I. The relative amounts of mRNA were calculated by relative standard-curve method, using ribosomal RNA as an endogenous control [25]. Figure 2b shows ratios of GFP mRNA relative to the value without co-expression of the LEA-like peptide. By means of two-side t-test, these relative ratios were shown to be identical, except for case of co-expression with the peptide with six repeating units, which showed a decrease in the relative value. These results confirmed that LEA-like peptide does not activate transcription of mRNA in *E.coli* and suggest, therefore, that co-expression with LEA-like 11-amino acid peptide effectively inhibits the aggregation of the unfolding target protein in the cytoplasm, and thereby resulting in increased protein expression.

When co-expressed with LEA-like peptides consisting of two to six repeating units, the expression of GFP decreased with increasing numbers repeat units in the peptide. This decrease may occur because the peptide suppresses the folding of the protein in the cytosol of *E.coli* or because there is a shortage in the cell of an amino acid required for the expression of the protein. Co-expression with peptide with six repeating units may have caused a decline in transcription because cell growth was affected by the co-expressed peptide.

### Validation of the Co-expression systems in protein expression

The expression of GFP was effectively increased by LEA-like peptide co-expression system in *E.coli*. In order to examine the versatility of the co-expression system, we evaluated two enzymes (*chloramphenicol acetyltransferase* (CAT), *Beta-glucuronidase* (GUS)) and one hydrophobic membrane protein (*hydrophobin:* HFBII) as our target protein for cellular expression by using LEA-like peptide co-expression system. CAT is a bacterial transferase that covalently attaches an acetyl group from acetyl-CoA to chloramphenicol. CAT forms a trimeric structure that is stabilized by a number of hydrogen bonds [26]. GUS is a glycosidase that catalyzes breakdown of complex carbohydrates. GUS exists as a homotetramer in cell [27]. These enzymes are used as a reporter gene to monitor gene expression in various cells. HFBII is one of the hydrophobins that was cloned from filamentous fungi *Trichoderma reesei* [28], [29] [30]. The protein acts as an adsorbent between the surface of the fungal cells and the solid surface. The HFBII have a size of about 75 amino acids, and a unique structure as rigidly amphiphile [28]. The expression of GFP, CAT and GUS were effectively increased by LEA-like peptide co-expression system (Table 1). The effects of the co-expression on the protein expression are enhanced with the increasing the molecular size (GFP: 27kDa, CAT: 25kDa, GUS: 78kDa). But, GFP-HFBII expression did not have enough enhancement of the protein expression by co-expression of LEA-like peptide. Bacterial expression of HFBII is essentially difficult, therefore, the effect of LEA-like peptide co-expression on the HFBII expression was less than the other protein expression. In order to confirm the difference of effectiveness on protein expression, it would be required further investigation by expression level of various type and size of protein using the LEA-like peptide co-expression system.

**Table 1:** The effects of the co-expression of LEA-like peptide on the protein expression.

	Relative protein expression after induction	
Protein	4 hour	8 hour
GFP	1.94	1.89
CAT	1.37	1.61
GUS	1.80	2.33
GFP-HFBII	1.26	1.20

Relative protein expression is the ratio of protein expression with LEA-like peptide to protein expression without LEA-like peptide. The molecular mass of protein is GFP: 27 kDa, CAT: 25 kDa, GUS: 78 kDa, and GFP-HFBII: 36 kDa, respectively.

### Function of the LEA-like peptide in protein expression

**Figure 3:**
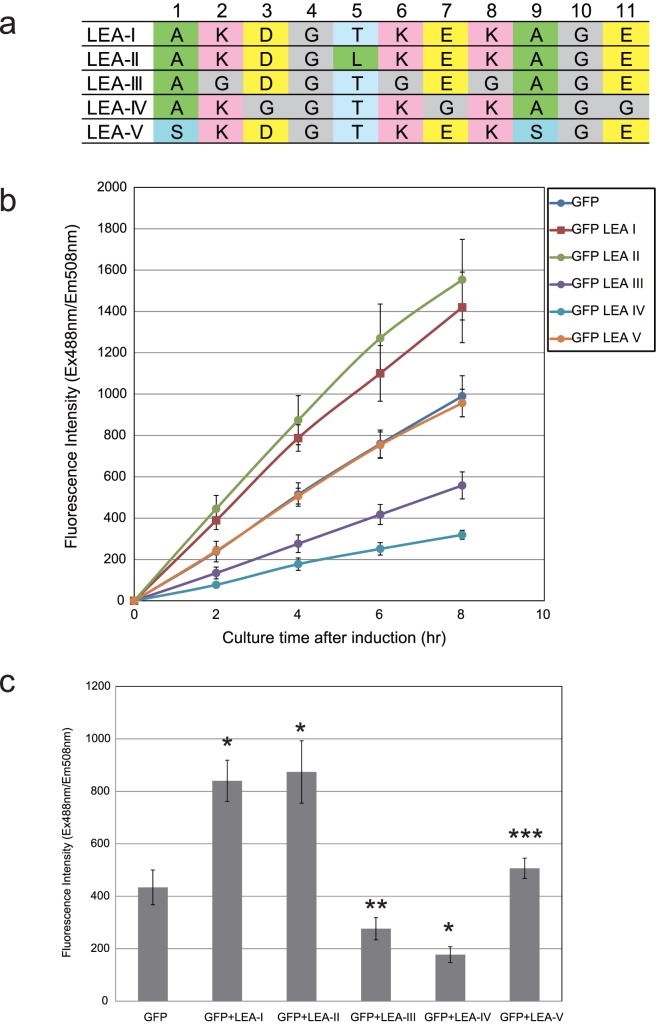
GFP expression with co-expression of each LEA-like peptide. (a) The designed sequence of each LEA-like peptide (b) Time dependence of GFP expression with co-expression of each LEA-like peptide (c) GFP expression with co-expression of each LEA-like peptide 4 h after induction. Data are expressed as mean ± s.d. (n = 5). Results with * P<0.0001 and ** P<0.005, shown above the bar were significantly different from those for GFP alone (two-side *t*-test). *** did not show significant difference from GFP alone (two-side t-test). Each P value is LEA-I: 0.0000214, LEA-II: 0.0000897, LEA-III: 0.0020771, LEA-IV: 0.0000486, and LEA-V: 0.8133212, respectively.

The repeating 11-amino acid sequence in Group 3 LEA proteins has characteristic motif that is observed in organisms as diverse as nematode, and eubacteria [20],[22],[24]. The motif is characterized by hydrophobic residues at position 1, 5 and 9; negative charged residues at positions 3, 7 and 11; and positive charged residues at positions 2, 6 and 8. A random assortment of amino acids are found in the other position (4 and 10), and these are not involved in the function of the LEA protein. To investigate the function of co-expressed LEA like peptide in the cell, we designed various types of peptides based on the LEA-like peptide. The amino acid sequences of these peptides are shown in Figure 3a. LEA-like peptide II (LEA-II) contains a hydrophobic amino acid (L-lucien;L) in the 5-position of the original LEA-like peptide I (LEA-I). In the LEA-like peptides III and IV (LEA-III and LEAIV), basic and acidic amino acid residues, respectively, were replaced by glycine (G) residues. In LEA-like peptide V (LEA-V), hydrophobic alanine (A) residues in the sequence were replaced by a hydrophilic amino acid (L-serine; S). Figure 3b shows the time course of GFP expression after induction when each of the LEA-like peptides was co-expressed. The protein expression increased slightly when LEA-II was co-expressed compared with co-expression with LEA-I. In contrast, the expression of GFP was markedly reduced when peptides with fewer charged groups were co-expressed. Positively and negatively charged amino acids in the peptide are therefore essential for boost protein expression by co-expression of LEA-like peptides. On the other hand, GFP expression was unaffected by co-expression of LEA-V, and the protein expression level was same that achieved in absence of the LEA-like peptide, according to a two-side *t*-test (Figure 3c). These results show that the presence of both the charged and hydrophobic amino acids in the sequence is necessary to boost protein expression by co-expression of the LEA-like peptide. From these results, we hypothesize that hydrophobic residues of LEA-like peptide attach to the protein surface and that the charged residues inhibit protein aggregation within the cell by mean of their electrostatics charges. To elucidate the mechanism of this phenomenon, it will be necessary to study the dependence on the peptide sequence and timing of the induction for expression of LEA-like peptide, adaptability for various types of the expressed protein, and the protein folding with this LEA-like peptide *in vitro*.

To achieve efficient expression of a protein, conventional methods are generally applied, such as the use of multicopy plasmids, enhancement of promoter activity, or expression with a hydrophilic protein tag. However, such conventional genetic methods are not sufficiently versatile for all types of expressed proteins and host cells, and tags have to be removed by protease digestion after expression. In contrast, the present elevation of protein expression by using an LEA-like peptide is highly versatile. This method is simple, in that the protein is co-expressed with only an 11-amino acid peptide. In addition, the separation process for protein purification is extremely simple, because the co-expressed peptide is very small. Therefore, these results could have a considerable impact, not only in applications involving bacterial protein expression, but also in relation to molecular biology.
